# Supplementary material for: ProteinShader: illustrative rendering of macromolecules
Source: BMC Struct Biol. 2009 Mar 30;9:19. doi: 10.1186/1472-6807-9-19 (PMC2672931; doi:10.1186/1472-6807-9-19)
Supplement: Additional file 1 — ProteinShader program without source code. This compressed file contains the complete ProteinShader program including associated libraries, but no source code. A README.txt file gives an overview of the ProteinShader distribution, and the index.html file in the help subdirectory has directions on getting started with the program as well as a set of tutorials. [file 1472-6807-9-19-S1.zip › ProteinShader-beta-0_9_4-binary/help/api/org/proteinshader/graphics/textures/package-use.html]

Uses of Package org.proteinshader.graphics.textures (ProteinShader API)


|  |  |  |
| --- | --- | --- |
| **Overview** \| **Package** \| Class \| **Use** \| **Tree** \| **Deprecated** \| **Index** \| **Help** | |  |
| PREV   NEXT | **FRAMES**    **NO FRAMES**     **All Classes** |  |


---


## **Uses of Package org.proteinshader.graphics.textures**

| Packages that use org.proteinshader.graphics.textures | |
| --- | --- |
| **org.proteinshader.graphics.adapter** | Holds the StructureToGraphics class, which is used to manage the use of the drawing classes and OpenGL display lists. |
| **org.proteinshader.graphics.textures** | Holds the classes needed for creating and managing OpenGL texture objects. |
| **org.proteinshader.gui** | Holds all of the Swing GUI components and their associated listeners, including class Renderer, which is registered as a listener for the GLCanvas object that is used a drawing surface. |

Classes in org.proteinshader.graphics.textures used by org.proteinshader.graphics.adapter

****Texture****
  
          Stores information on an OpenGL texture object.

Classes in org.proteinshader.graphics.textures used by org.proteinshader.graphics.textures

****ConfigReader****
  
          Reads a configuration file to obtain a list of menu names
and matching filenames for textures.

****NamePair****
  
          Stores a menu name and a file name for a texture.

****Texture****
  
          Stores information on an OpenGL texture object.

Classes in org.proteinshader.graphics.textures used by org.proteinshader.gui

****Texture****
  
          Stores information on an OpenGL texture object.

---


|  |  |  |
| --- | --- | --- |
| **Overview** \| **Package** \| Class \| **Use** \| **Tree** \| **Deprecated** \| **Index** \| **Help** | |  |
| PREV   NEXT | **FRAMES**    **NO FRAMES**     **All Classes** |  |


---

# *Copyright © 2007-2008*
